# Cardiac magnetic resonance imaging: artefacts for clinicians

**DOI:** 10.1007/s12471-014-0623-z

**Published:** 2014-10-23

**Authors:** A. W. M. van der Graaf, P. Bhagirath, S. Ghoerbien, M. J. W. Götte

**Affiliations:** Department of Cardiology, Haga Teaching Hospital, Leyweg 275, 2545 CH The Hague, the Netherlands

**Keywords:** Cardiac magnetic resonance, Image artefacts, MR physics, Image optimisation

## Abstract

In recent years, the clinical importance of cardiac magnetic resonance (CMR) imaging has increased dramatically. As a consequence, more clinicians need to become familiar with this imaging modality, including its technical challenges. MR images are obtained through a physical process of proton excitation and the reception of resonating signals. Besides these physical principles, the motion of the heart and diaphragm, together with the presence of fast flowing blood in the vicinity, pose challenges to the acquisition of high-quality diagnostic images and are an important cause of image artefacts. Artefacts may render images non-diagnostic and measurements unreliable, and most artefacts can only be corrected during the acquisition itself. Hence, timely and accurate recognition of the type of artefact is crucial. This paper provides a concise description of the CMR acquisition process and the underlying MR physics for clinical cardiologists and trainees. Frequently observed CMR artefacts are illustrated and possible adjustments to minimise or eliminate these artefacts are explained.

## Introduction

Cardiac magnetic resonance (CMR) imaging is the current gold standard for the assessment of left and right ventricular function and the quantification of intramyocardial fibrosis [1, 2]. Cardiac and respiratory motion, together with fast flowing blood in the vicinity, challenge the acquisition of high-resolution, diagnostic images. In addition, adjacent tissues with different magnetic characteristics or the presence of implants may cause local loss of signal. These phenomena and their visual consequences are referred to as artefacts [3, 4].

Artefacts can severely degrade the quality of acquired images and may render images non-diagnostic and measurements unreliable. Most artefacts can only be corrected during the scan procedure itself. Therefore, timely recognition of the type of artefact and proper adaptation of the acquisition strategy are essential.

In recent years, the number of clinical cardiologists involved in the acquisition and reporting of CMR images has increased substantially [5]. Hence, knowledge of everyday CMR artefacts is important. In order to comprehend the origin of these artefacts, basic familiarity with MR physics is required.

This paper provides a concise and practical description of the CMR acquisition process and the underlying MR physics for clinical cardiologists and trainees. Most frequently observed CMR artefacts are illustrated and explained, and methods to eliminate the artefacts are suggested.

## MR physics

An MR system consists of the main magnet, three gradient coils and an integral radiofrequency (RF) transmitter. The main magnet generates a strong, constant magnetic field (measured in Tesla). A field strength of 1.5 T is commonly used in CMR. By gradually altering the strength of the main magnetic field along the gradient axis, the gradient coils are used for slice selection (z axis) and encoding of signals in the frequency (x axis) and phase (y axis) encoding direction. These frequency and phase encoding directions can be regarded as the x and y axis of a 2-dimensional graph. This graph represents the image matrix. A higher spatial resolution can be achieved by decreasing the slice thickness and increasing the number of frequency and phase encoding steps.

Upon entering the MR magnet bore, all hydrogen protons in the body either align parallel or anti-parallel with the static magnetic field. During the scan, the integral RF transmitter excites (flips) a selected group of hydrogen protons over a predefined angle (flip angle). After cessation of the RF pulse, the protons return to their original state. T1 (along the z axis) and T2 (in the xy plane) relaxation times are used to describe this recovery process. Because all tissues have characteristic T1 and T2 relaxation times, organs and structures can be distinguished based on their particular signal intensity. T1- or T2-weighted imaging sequences use this feature to visualise the difference in signal intensity between varying types of tissues.

Receiver coils, either positioned on the patient’s body or mounted inside the MR bore, collect the signals from resonating protons. The raw signal data are subsequently stored in the K-space, a virtual temporary memory. After a mathematical transformation (Fourier transform), an image can be generated [6].

## Pulse sequence

A programmed run of repetitive pulses and switching of gradients is called a *pulse sequence*. Various pulse sequences can be used, depending on the type of imaging desired. The *spin echo* sequence (using a 90° flip angle) and the *gradient echo* sequence (flip angle between 0 and 90°) are frequently used. A greater flip angle usually results in a higher signal. In CMR, spin echo sequences are frequently used for anatomic imaging. Gradient echo sequences allow more rapid acquisition and are often used for function or flow imaging [7].

## Important parameters in CMR

- The *field of view (FOV)* defines the area of interest and should preferably be positioned in the centre of the magnet bore (isocentre), where the magnetic field is highly homogeneous. The FOV is the total area covered by the matrix of the frequency and phase encoding direction. It is very important to ensure that the structure or region of interest is enclosed by the selected FOV.
- The *repetition time (TR)* defines the time between two sequential RF pulses within a pulse sequence. After the initial RF pulse, additional pulses can be applied to refocus the resonating signal and create a so-called echo of the initial signal. The *echo time (TE)* marks the time from the RF pulse to the moment at which the signal of the echo is at its highest point. Together, the TR and TE determine the level of T1- or T2- weighting of an image.
- The *inversion time (TI)* represents the period between the inversion pulse (180°) and the excitation pulse. In sequences used for late gadolinium enhancement (LGE) imaging, it is important that the excitation pulse is timed when the signal from the healthy myocardium passes the zero point (‘nulled’). When the signal passes the zero point, there is no signal to obtain from the resonating protons and the healthy myocardium will appear black. In this way, the difference in signal intensity between healthy and abnormal myocardium can be observed. This difference can be maximised by the use of gadolinium-based contrast agents.

## Specific absorption rate

The specific absorption rate (SAR) defines the RF power absorbed per unit of mass [8]. The SAR is measured in watt per kilogram (W/kg). Absorption of electromagnetic energy results in the generation of heat. The whole body SAR is restricted to 4 W/kg, the maximum SAR for the thorax is 8 W/kg [9]. When implanted devices are present, the SAR is usually restricted to 2 W/kg [10]. General measures to limit the SAR include reduced scan time, use of a lower flip angle and a longer TR.

Before referring a patient for CMR, the absence of any contraindications and the ability to lie down for at least 30–45 min should be confirmed. It is best to explain the procedure and practice breath-holding prior to the exam.

Because data acquisition is synchronised to the R wave in the QRS complex, an optimised ECG signal is essential for obtaining high-quality images.

## Artefacts

The most important characteristics of the artefacts and their possible solutions are summarised in Table 1.

**Table 1:** Characteristics of common CMR artefacts

Artefact	Reason	Appearance	Solution	Penalty
1. Aliasing	Field of view (FOV) does not enclose all body parts	Region outside FOV is projected on other side of the image	- Increase FOV / use oversampling in phase encoding direction	- Increased scan duration
			- Swap phase and frequency direction	- Possible aliasing
			- Apply saturation band on body part that is projected	- Slightly increased scan duration
1.1. Parallel imaging	Undersampling	Region outside FOV is projected in the image	- Decrease parallel imaging factor - Increase FOV	- Increased scan duration - Increased scan duration
1.2. Aliasing in flow sequence	Velocity encoded on the scanner (VENC) does not match the true velocity in the vessel	Dark region (black holes) inside vessel in flow series	- Increase or decrease the VENC	- No penalty
2.1 Motion: Ghosting	- Respiratory motion - Movement / pulsation	Parallel lines or contours	- Breath-hold instruction / practice with patient - Breath-hold on inspiration / expiration?	- No penalty - Degree of inspiration is more variable
			- Use single-shot imaging	- Reduced image quality
			- Decrease spatial resolution	- Reduced image quality
			- Navigator-gated	- Increased scan duration
			- Real-time acquisition	- Increased scan duration, reduced image quality
			- Swap phase and frequency direction	- No penalty
			- Apply saturation band on body part that is projected	- Increased scan duration
2.2 Motion: Trigger	- Poor ECG signal - Arrhythmia	Blurry myocardial borders	- Optimize ECG signal / check electrode position	- No penalty
			- Use single-shot imaging	- Decreased spatial resolution
			- Select arrhythmia rejection on scanner	- Increased scan duration
			- Prospective triggering	- Incomplete imaging of heart cycle
			- Real-time acquisition	- Increased scan duration, reduced image quality
3. Blood flow	Nearby flowing protons disturb local magnetic field	Flow-related distortion of specific region	- Shimming - Reduce repetition time/echo time - Apply saturation band - Swap phase and frequency direction - Adjust slice selection	- No penalty - No penalty - Slightly increased scan duration - Possible aliasing - No penalty
4. Radiofrequency (RF)	Interference of an external RF source with the MR magnetic field	Regular striped pattern across all images	- Remove RF source - Close door MR room	- No penalty - No penalty
5. Chemical shift	Different resonance frequencies of water and fat protons in one voxel	Signal void at anatomic intersections	- Increase bandwidth (increase slice thickness) - Apply fat suppression	- Decreased spatial resolution - Increased scan duration
6. Dark rim	Difference in high and low signal causes signal void	Dark (sub)endocardial ring	- Increase spatial resolution	- Increased scan duration
7. Inhomogeneity	Dephasing of spins	Signal void or circles/blooming	- Shimming - Use (fast) spin echo instead of gradient echo - Apply saturation band on implant	- No penalty - Increased scan duration - Slightly increased scan duration and SAR

## Aliasing

**Fig. 1:**
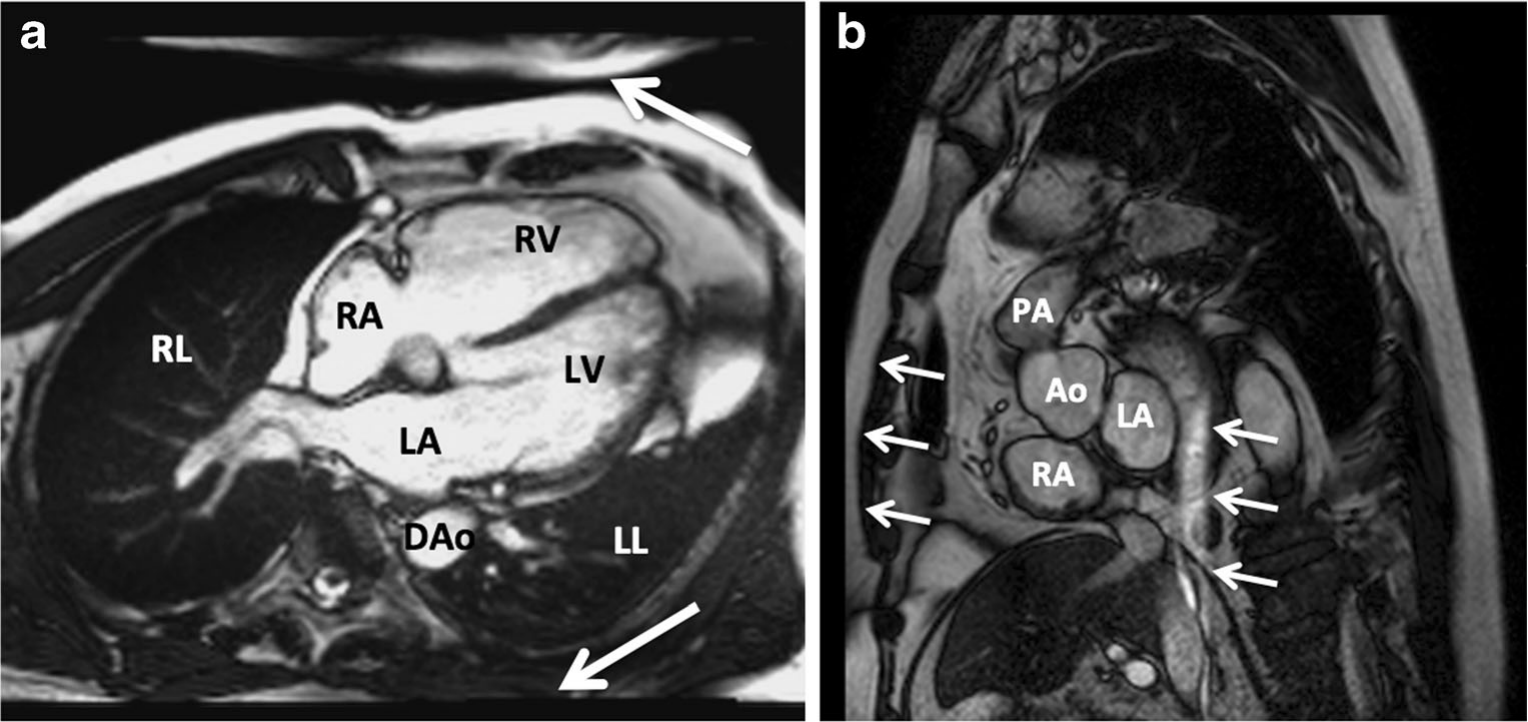
Steady-state free-precession (SSFP) cine images can be used for function analysis. Four-chamber view with signal averaging of the aorta. The region outside the field of view (FOV) wraps around at the other side of the image (*white arrows*) (**a**). When using parallel imaging and an acceleration factor of two, the region outside the FOV wraps around in the middle of the image (**b**). *RA* right atrium; *RV* right ventricle; *LA* left atrium; *LV* left ventricle; *DAo* descending aorta; *LL* left lung; *RL* right lung; *PA* pulmonary artery; *Ao* aorta

Aliasing occurs when the FOV does not enclose all body parts being imaged [11]. The region outside the FOV wraps around and is projected at the opposite side of the image (Fig. 1a). The projected body part may cover the area of interest.

This artefact is often resolved by increasing the FOV or the number of phase encoding steps (oversampling). Another option is to swap the frequency and phase encoding direction in the acquisition process. Regarding the asymmetry of the torso, the number of phase encoding steps may be adequate for acquisition in the opposite direction. If the aliasing persists, the body part may be projected outside the area of interest.

Saturation bands involve the application of an inversion pulse on a specified area. When a saturation band is targeted on the body part outside the FOV, the signal from this particular area is inverted and can no longer cause aliasing.

### Parallel imaging artefact

Parallel imaging is a technique that reduces the acquisition time by undersampling in the phase encoding direction. Parallel imaging uses the information about the local sensitivity of each coil element. The FOV is intentionally made too small in the phase encoding direction, and the resulting aliasing is unwrapped using this information [12, 13]. The selected acceleration factor defines the extent of undersampling performed. For example, when the acceleration factor is two, only half of the available K-space is used. However, the signal to noise ratio is often reduced at higher acceleration factors.

When aliasing occurs, the region outside the FOV will be projected in the region of interest. By reducing the parallel imaging (acceleration) factor, the artefact will be forced to the borders of the image (Fig. 1b). Alternatively, the FOV can be increased to enclose all body parts.

### Aliasing in flow series

In flow sequences, the phase shift of moving hydrogen protons is observed. This phase shift is proportional to the velocity of flowing protons. The encoded velocity (VENC) is a parameter on the scanner that represents the maximum velocity present in the imaging volume. Any velocity greater or smaller than the preselected VENC causes aliasing.

**Fig. 2:**
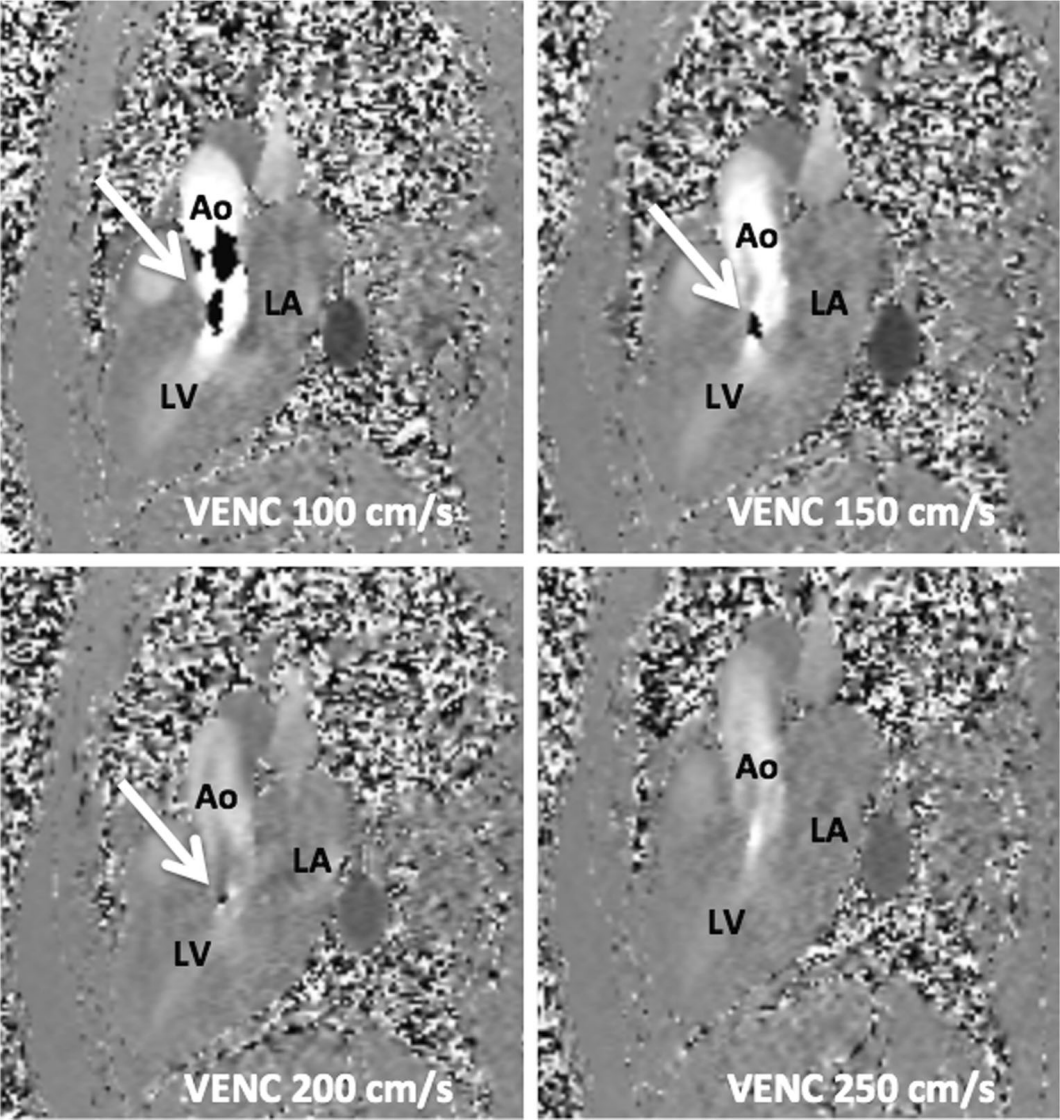
Aliasing in flow sequences in a patient with hypertrophic cardiomyopathy and turbulence in the left ventricular outflow tract. The aliasing artefact decreased and later vanished upon elevation of the velocity-encoding (VENC) gradient

Aliasing appears as black holes (Fig. 2) in the flow sequence [14]. It is important to detect this artefact, since it will lead to underestimation or overestimation of the true velocity. The VENC should be manually adjusted, until the velocity encoded on the scanner slightly exceeds the velocity in the body of the patient. Correct adjustment of the VENC will eliminate the artefact.

## Motion artefacts

### Ghosting

**Fig. 3:**
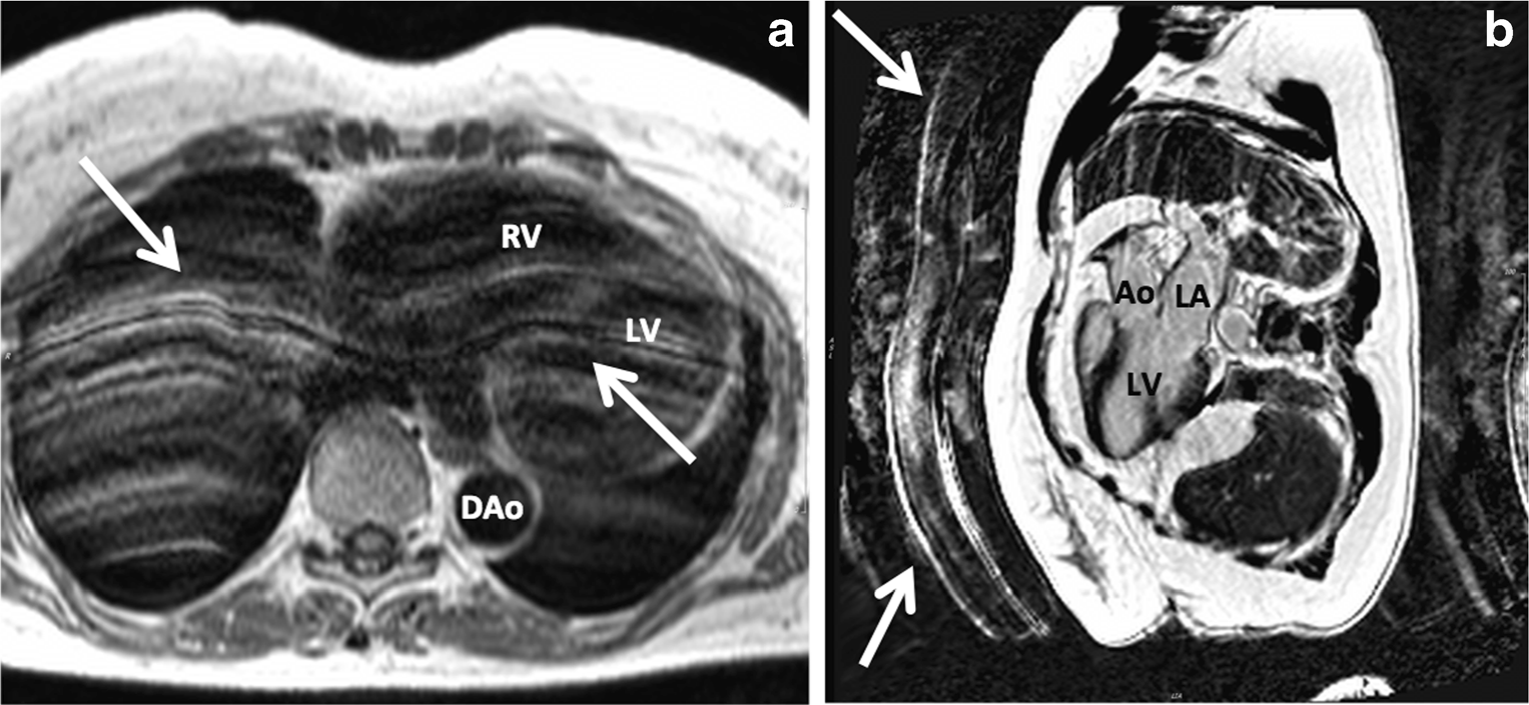
Ghosting artefact caused by respiratory motion in a Turbo Spin Echo T1 weighted (*black blood*) sequence, axial view (**a**) and phase sensitive inversion recovery (PSIR) LGE sequence, 3-chamber view (**b**)

Ghosting refers to the appearance of parallel lines or double contours in the image (Fig. 3). These are often repeated projections of the abdominal or chest wall. This artefact is most often caused by respiratory motion during the acquisition [15].

Measures to eliminate the ghosting artefact primarily concern controlled breathing during the acquisition and coaching of the patient. When the patient is unable to perform breath-holds in expiration, breath-hold in inspiration can be used.

Single shot imaging or reducing the spatial resolution can be used to speed up the acquisition and decrease the breath-hold duration.

A navigator sequence can be used to monitor the movement of the diaphragm during free breathing. Image acquisition is synchronised to the diaphragmatic excursions. Navigator-gated imaging can be time-consuming, because data acquired outside a pre-set acceptance window are rejected [16]. Alternatively, real-time imaging does not require breath-holds and is not ECG triggered. It is suitable for patients with difficulty in performing breath-holds or in case of arrhythmia. However, the acquisition time will increase and the image quality decreases severely. The signal from the abdominal wall may be inverted by the application of a saturation band.

### Trigger artefact

Cardiac data acquisition is synchronised to the R wave in the QRS complex. Normally, data are collected during the complete heart cycle and retrospectively assigned to specific phases of the cardiac cycle (retrospective triggering). In the presence of a poor ECG signal or arrhythmia, data acquisition may become challenging. When a trigger artefact is present, myocardial borders become less well defined or blurry. The image quality may decline and render the examination non-diagnostic and the measurements or calculations performed unreliable.

Arrhythmia rejection is a software option that can be used in patients suffering from an irregular heartbeat. Images obtained during irregular RR intervals are rejected.

Prospective triggering is an alternative strategy in which data are acquired during a predefined period of the cardiac cycle. An important limitation of this approach is the fact that image acquisition does not cover the complete RR interval. Hence, the stroke volume may be underestimated in the volume analysis.

In case of severe arrhythmia or difficulty to perform breath-holds, real-time imaging can be used. During real-time imaging data acquisition is no longer synchronised to the R wave, but is continuous for a preselected time period. In this way, ventricular function and wall motion abnormalities can be globally assessed. However, real-time imaging prolongs the scan duration and often results in images with a severely decreased spatial resolution.

### Blood flow artefact

**Fig. 4:**
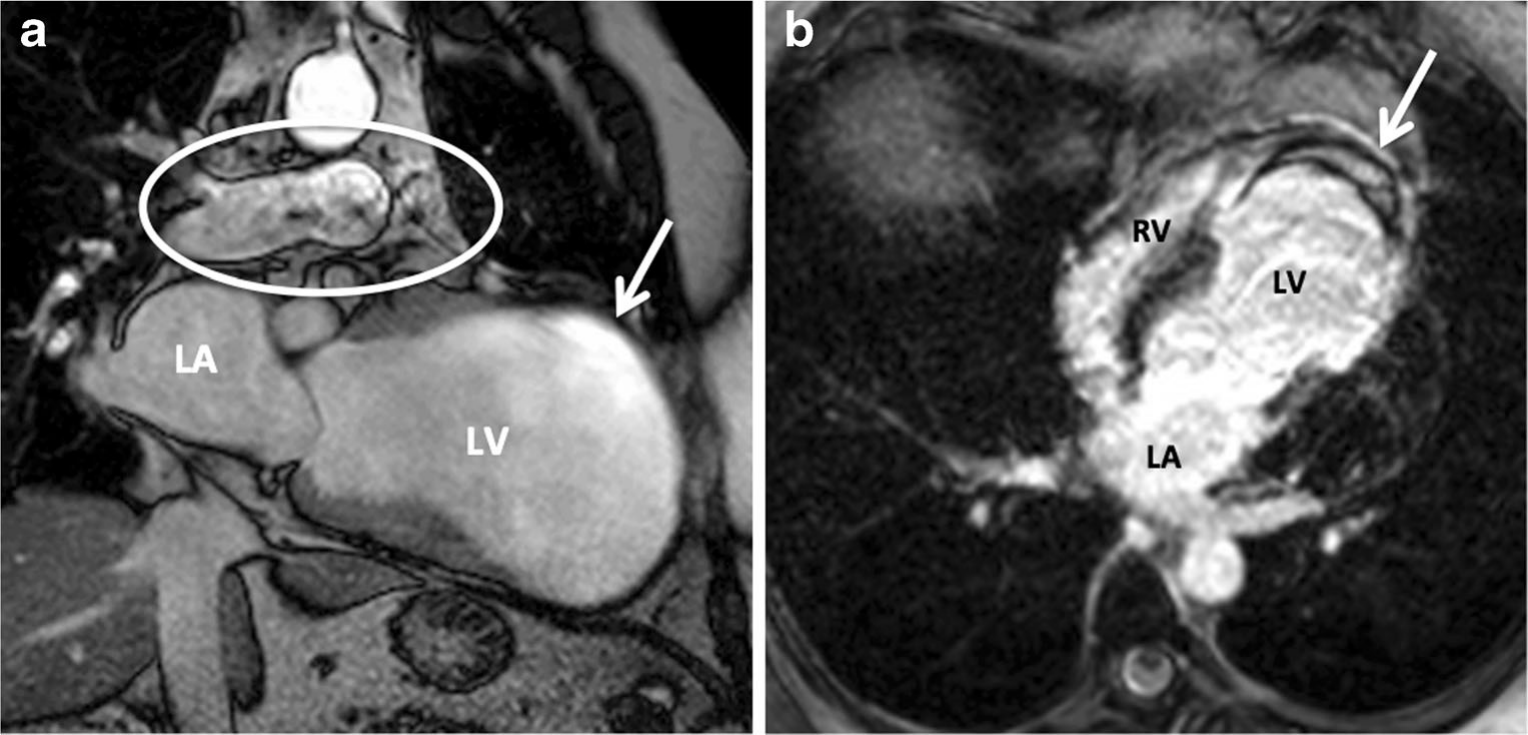
Long-axis (two-chamber) view of the heart of a patient with an old infarction in the LAD trajectory and a large aneurysm of the anterior wall. During systole (**a**), a flow artefact (*encircled*) caused by the blood flowing through the pulmonary artery can be observed. The white arrow in panel **a** indicates an inhomogeneity artefact. Inhomogeneity artefacts usually arise when structures with different magnetic properties coexist in a small area. The white arrow in panel **b** indicates a chemical shift artefact, surrounding a thrombus in the LV apex

Protons flowing at a high velocity near or in the selected imaging slice can disturb the homogeneous steady state magnetisation [17]. This occurs when the area of interest is close to outflow tracts or large arteries (Fig. 4a).

To overcome this problem, the homogeneity of the main magnetic field can be locally improved (shimming). Reducing the TR or TE results in a sequence less susceptible for turbulent flow artefacts. Alternatively, the signal from the passing protons may be inverted by the application of a saturation band across the outflow tract or artery. By swapping the phase and frequency encoding direction, the artefact may be projected outside the region of interest. Finally, slice selection may be adjusted.

## Radiofrequency / Zipper artefact

An RF artefact is caused by distortion of the magnetic RF field by an external RF source. It is characterised by a regular striped pattern across all images, irrespective of the MR sequence used. This artefact may arise when the door of the MR suite is not properly closed or when the isolation of the MR room (Faraday cage) is damaged.

## Chemical shift artefact

Chemical shift artefacts appear at the interfaces between fat and water-based tissues [18]. There are two kinds of chemical shift artefacts. The first kind is caused by a misregistration of the signal from fat and water protons present in the same voxel along the frequency encoding direction. The difference in resonance frequency between fat and water causes a separation (pixel shift) in the reconstructed images. The degree of pixel shift depends on the receiver bandwidth used. In general, a higher bandwidth is associated with a smaller pixel shift. The second kind is caused by dephasing of fat and water protons and causes signal cancellation (Fig. 4b).

It is generally hard to eliminate this artefact. By increasing the bandwidth the artefact can be diminished. The difference between the frequency of fat and water protons in one voxel is reduced. Alternatively, the signal from fat can be suppressed through the application of a fat suppression sequence.

## Dark rim artefact

**Fig. 5:**
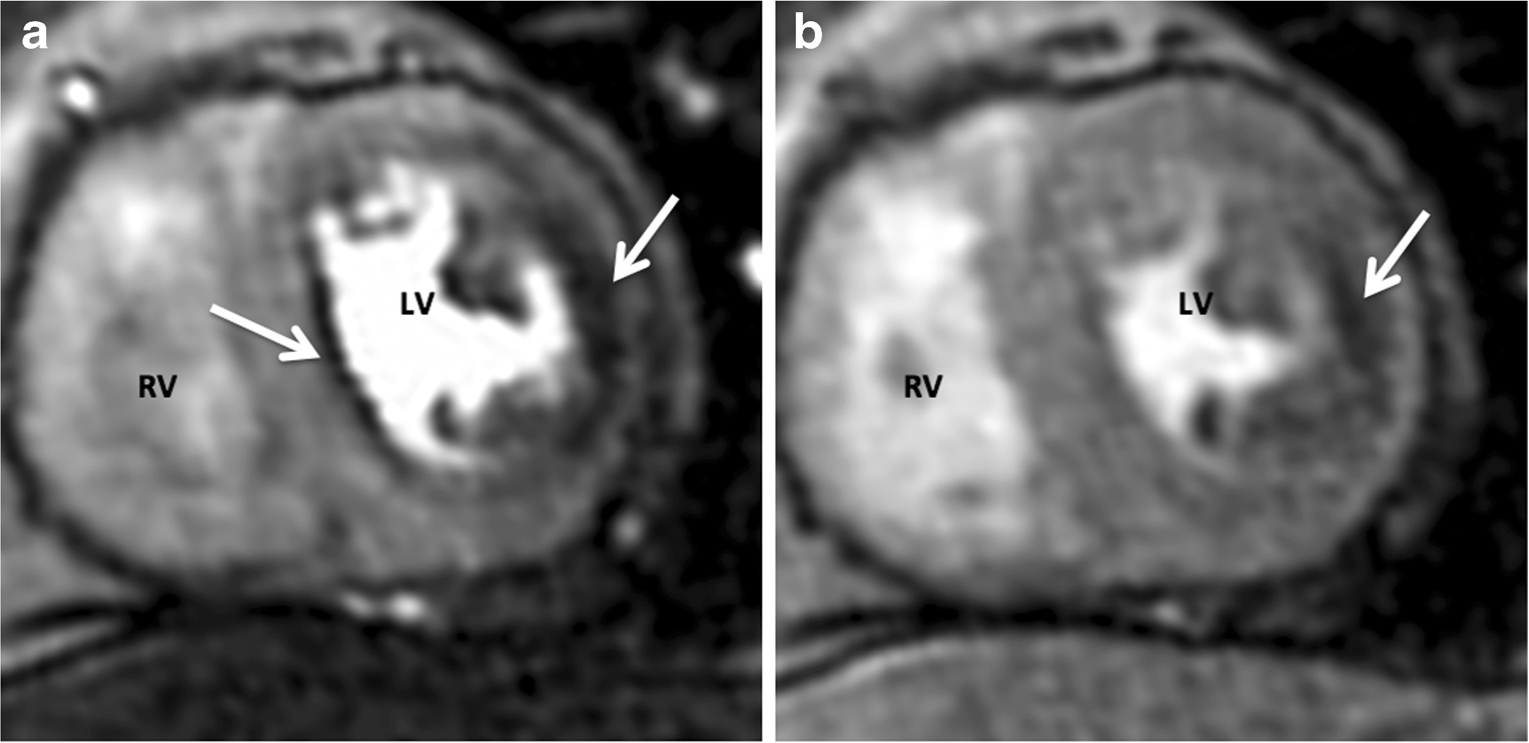
Comparison between a Gibbs ringing artefact (*septal wall*) and a true subendocardial perfusion defect (*lateral wall*) in the same patient. The artefact usually lasts for a few heartbeats (**a**). A perfusion defect tends to be more persistent (**b**)

The Gibbs ringing artefact is a frequent cause of dark rim artefacts. Gibbs artefacts can be observed in any CMR image at the intersection of a bright (blood pool) and darker (myocardium) signal [19]. In CMR perfusion imaging, a dark rim artefact may be hard to differentiate from a subendocardial perfusion defect (Fig. 5).

Increasing the spatial resolution can reduce this artefact. However, presence of the artefact does not have to prohibit image analysis for experienced image readers. Even in the presence of a dark rim artefact, a true perfusion defect can be discriminated. The artefact usually lasts for only a few heartbeats, while a real perfusion defect tends to be more persistent.

## Inhomogeneity artefacts

Due to the magnetic properties of most body tissues, the applied magnetic field is slightly disturbed (inhomogeneous). This phenomenon causes regional dephasing of protons at the boundaries between different tissues, particularly between muscle and lung [20].

**Fig. 6:**
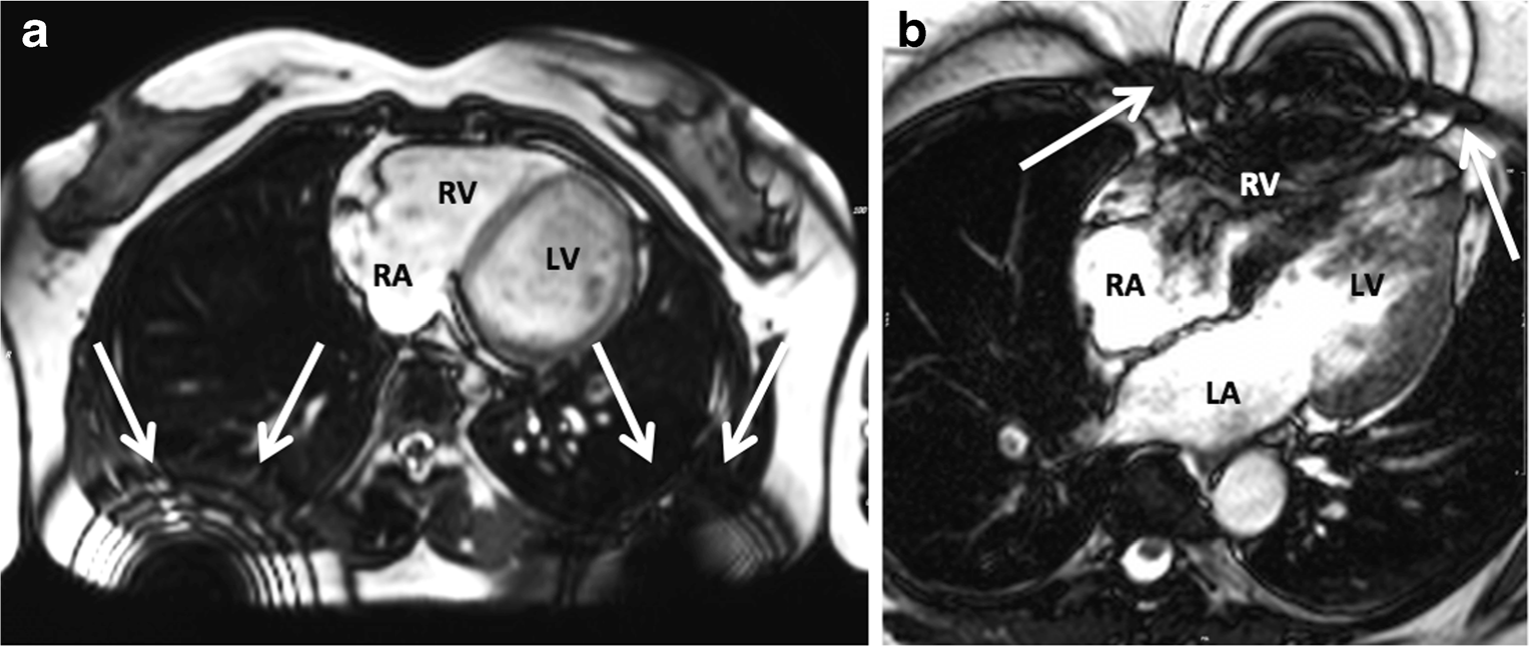
Example of susceptibility artefacts caused by the presence of ferromagnetic materials. In panel **a** the artefact is caused by the presence of a bra. In panel **b**, an implanted loop recorder was present during the examination

Alternatively, this artefact may arise from the presence of an implanted foreign body (metallic artefact) (Fig. 6a and b). Metallic susceptibility artefacts tend to worsen at a higher magnetic field strength. Apart from safety measures, it may therefore be advisable to use a scanner of limited field strength (1.5 T) in patients with coronary stents, implanted devices or prosthesis.

Intermittent use of a 180° refocusing pulse in the applied pulse sequence may rephase the protons. For this reason, spin echo sequences are less sensitive to susceptibility artefacts than gradient echo sequences. A saturation band may be used to suppress the signals from the implant. Shimming can eliminate local inhomogeneities in the static magnetic field of the MR.

There are two types of shimming: active and passive. Passive shimming is performed during installation of the MR scanner. The MR operator can perform active shimming using dedicated shim coils.

## Take home message

Artefacts can severely degrade the diagnostic quality of CMR images. However, with limited knowledge of the underlying physics and basic familiarity with common CMR artefacts, the type of artefact should be recognisable. This is important because most artefacts require immediate intervention during the scan procedure.

## Conclusion

Artefacts can severely degrade the diagnostic quality of CMR images, but may be easily resolved by adjusting certain parameters or conditions. This educational article attempts to facilitate the recognition and understanding of common CMR artefacts by clinicians and trainees. This has become relevant, because an increasing number of cardiologists are involved in the acquisition and reporting of CMR images.
